# Memory T cells skew toward terminal differentiation in the CD8+ T cell population in patients with acute myeloid leukemia

**DOI:** 10.1186/s13045-018-0636-y

**Published:** 2018-07-09

**Authors:** Ling Xu, Danlin Yao, Jiaxiong Tan, Zifan He, Zhi Yu, Jie Chen, Gengxin Luo, Chunli Wang, Fenfang Zhou, Xianfeng Zha, Shaohua Chen, Yangqiu Li

**Affiliations:** 1 Department of Hematology, First Affiliated Hospital, Institute of Hematology, School of Medicine; Key Laboratory for Regenerative Medicine of Ministry of Education, Jinan University, No.601 West of Huangpu Avenue, Guangzhou, 510632 China; 2 Department of clinical laboratory, First Affiliated Hospital, Jinan University, Guangzhou, 510632 China

**Keywords:** Stem cell memory T cells, Central memory T cells, Effector memory T cells, CD8+ T cells, Acute myeloid leukemia, Bone marrow, Peripheral blood

## Abstract

Stem cell memory T (T_SCM_) and central memory T (T_CM_) cells can rapidly differentiate into effector memory (T_EM_) and terminal effector (T_EF_) T cells, and have the most potential for immunotherapy. In this study, we found that the frequency of T_SCM_ and T_CM_ cells in the CD8+ population dramatically decreased together with increases in T_EM_ and T_EF_ cells, particularly in younger patients with acute myeloid leukemia (AML) (< 60 years). These alterations persisted in patients who achieved complete remission after chemotherapy. The decrease in T_SCM_ and T_CM_ together with the increase in differentiated T_EM_ and T_EF_ subsets in CD8+ T cells may explain the reduced T cell response and subdued anti-leukemia capacity in AML patients.

## To the editor

Clinical applications of immunotherapy for AML lag behind those for solid tumors and lymphocytic leukemia [1–3]. Recently, a new memory T cell subset, stem cell memory T (T_SCM_), which has stem cell-like capacity, has been discovered [4–6]. However, little is known about the role of these cells in AML. In this study, we assessed the distribution of CD4+ and CD8+ T_SCM_, central memory T (T_CM_), T effector memory (T_EM_), and T terminal effector (T_EF_) cells in peripheral blood (PB) and bone marrow (BM) from patients with AML and those with AML in complete remission (AML-CR) by multicolor flow cytometry. The gating strategy used in this study followed a published protocol [7]. The CD4+ and CD8+ T cells were divided into four subgroups according to the CCR7 and CD45RO expression pattern: naïve and T_SCM_ cells (CCR7+CD45RO−), T_CM_ cells (CCR7+CD45RO+), T_EM_ cells (CCR7−CD45RO+), and T_EF_ cells (CCR7−CD45RO−). The T_SCM_ population was defined by double positive CD95 and CD28 expression.

**Fig. 1:**
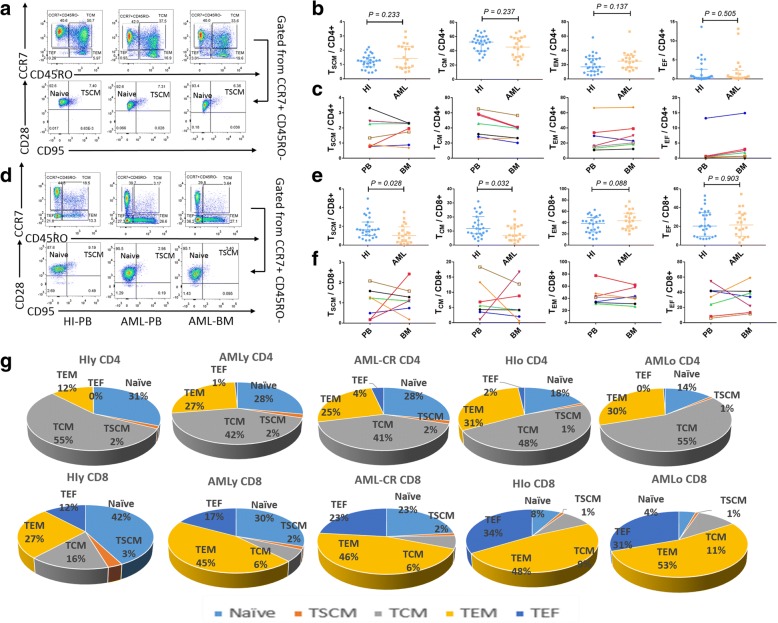
Gating strategy for identifying the CD4+ and CD8+ T cells and the percentage of memory T cell subsets in the patients with AML and healthy individuals. **a**, **d** CD4+ (**a**) and CD8+ T (**d**) cells were differentiated into four subsets based on the expression of CCR7 and CD45RO in one HI-PB, one AML-PB, and one AML-BM patient: central memory T cells (CCR7+CD45RO+), effector memory T cells (CCR7−CD45RO+), and effector T cells (CCR7−CD45RO−). In the CCR7+CD45RO− subset, the expression of CD28 and CD95 was used to identify naïve T cells (CD28+CD95−) and T_SCM_ cells (CD28+CD95+). **b**, **e** Frequency of the T_SCM_, T_CM_, T_EM_, and T_EF_ subsets in the CD4+ (**b**) and CD8+ (**e**) T cell populations from 27 HIs and 20 AML patients. **c**, **f** The subsets within the CD4+ (**c**) and CD8+ (**f**) T cell populations from PB and matched BM from seven AML patients, including different AML subtypes (M1, M2, M2b, M3, and M5), were compared. **g** Summary of the altered distributions within the CD4 and CD8 naive and memory T cell subsets in the AMLy, AMLo, and AML-CR groups compared with HIs. HIy (*n* = 13), AMLy (*n* = 10), AML-CR (*n* = 9), HIo (*n* = 14), AMLo (*n* = 10). HIs, healthy individuals; AML, acute myeloid leukemia; AML-CR, AML patients who achieved complete remission; PB, peripheral blood; BM, bone marrow; y, younger than 60 years; and o, older than 60 years. The differences in the different T cell populations in each of the T cell subsets were tested by two independent-sample Wilcoxon tests. Medians were calculated to represent all of the data. *P* values < 0.05 were considered statistically significant

The percentages of the T_SCM_, T_CM_, T_EM_, and T_EF_ cells in the CD4+ and CD8+ populations were analyzed in 20 cases with AML (17 cases in newly diagnosed and 3 cases with AML relapse) (Fig. 1a, d) [8, 9]. The CD8+ T_SCM_ and CD8+ T_CM_ cells significantly decreased in the PB of these patients (Fig. 1e, g), whereas there was no significant change in the CD4+ population (Fig. 1b, g). Thus, the changes in the memory T cell subsets appeared to mainly involve CD8+ T cells. The shift from T_SCM_ and T_CM_ cells to a higher ratio of differentiated T_EM_ and T_EF_ cells is thought to be due to the constant exposure of T cells to AML cells and the leukemia environment, leading to T cell exhaustion and/or dysfunction [3].

To study the influence of the tumor microenvironment on the memory T cell distribution and function in leukemia patients, we collected seven pairs of PB and BM samples from AML patients at the time of diagnosis and compared the distributions of memory T cell subsets. The differences in each subset appeared to vary widely (Fig. 1c, f). A low percentage of CD4+ T_CM_ cells and a corresponding high percentage of CD4+ T_EM_ and T_EF_ cells were observed in the BM compared with PB (Fig. 1c). In the CD8+ population, the changes appeared to be specific to each individual, and lower CD8+ T_SCM_ and CD8+ T_CM_ percentages were observed in the BM in half of the patients, whereas there were high percentages of CD8+ T_SCM_ and CD8+ T_CM_ cells in the BM compared with PB in the remaining samples. It has been reported that T cells in normal BM mainly possess a memory phenotype, particularly for CD8+ T_CM_ cells [10], suggesting that alterations in the leukemic BM niche in different AML individuals and AML subtypes may have different impact on T_CM_ homing.

**Fig. 2:**
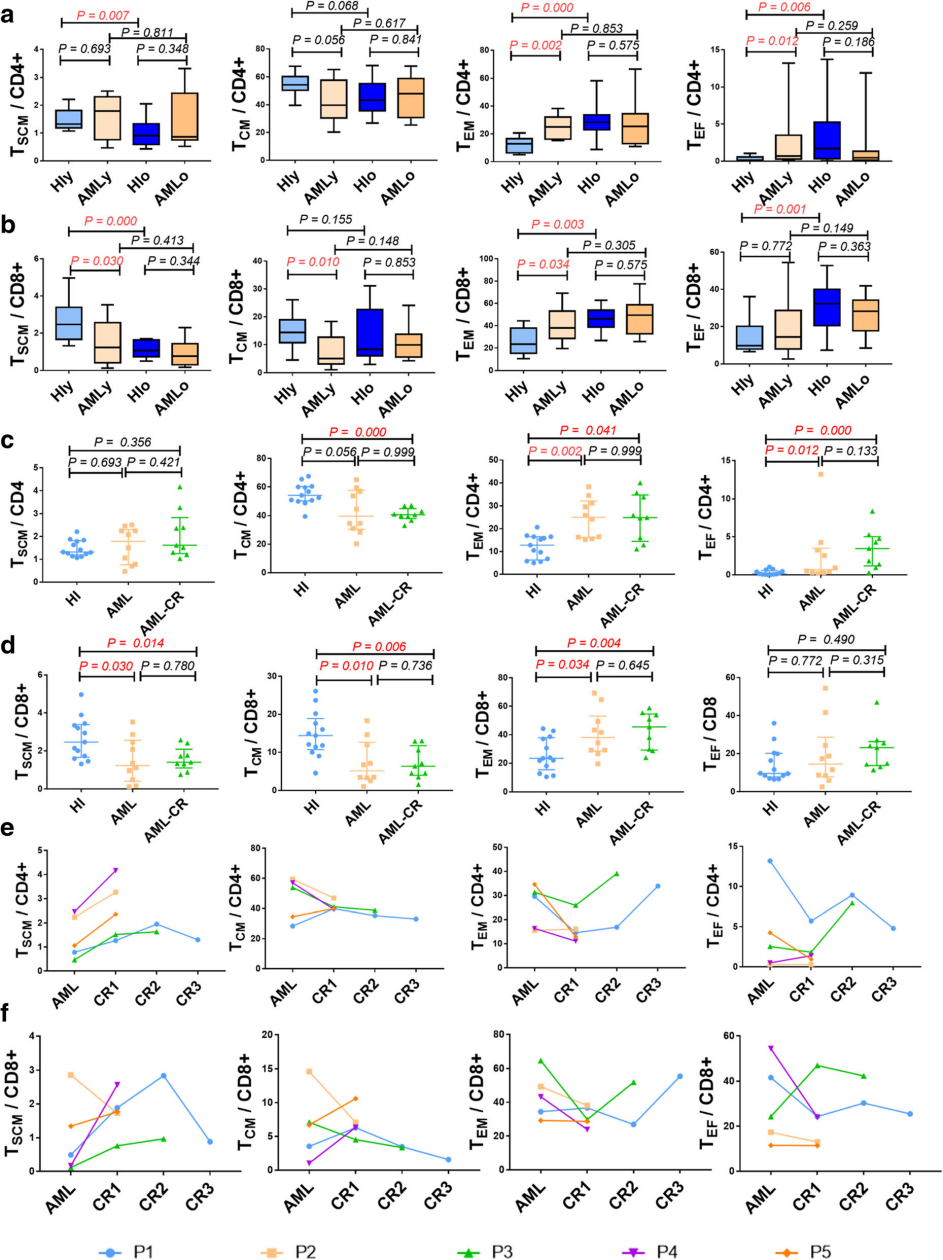
Memory T cell subset distribution in CD4+ and CD8+ T cells in patients younger or older than 60 years with AML and AML-CR. **a**, **b** T_SCM_, T_CM_, T_EM_, and T_EF_ subsets within the CD4+ (**a**) and CD8+ (**b**) populations in the HIy, AMLy, HIo, and AMLo groups. HIy (*n* = 13), AMLy (*n* = 10), HIo (*n* = 14), and AMLo (*n* = 10). **c**, **d**: Frequency of T_SCM_, T_CM_, T_EM_, and T_EF_ cells within the CD4+ (**c**) and CD8+ (**d**) T cell populations in age matched HI, AML and AML-CR cohorts. HIs (*n* = 13), AML (*n* = 10), and AML-CR (*n* = 10). **e**, **f** Five AML patients were dynamically assayed for the T_SCM_, T_CM_, T_EM_, and T_EF_ subsets in the CD4+ (**e**) and CD8+ (**f**) T cell populations at different time points. AML-CR, AML patients who achieved complete remission; P, patient; CR1, 2, 3, indicate different time points at which the patient achieved CR

Next, we compared the distribution of memory T cells in AML patients younger (AMLy) and older (AMLo) than 60 years [11]. Unlike healthy individuals (HIs), the memory T cell subset distribution in the AMLy cohort was strikingly different than that in younger HIs (HIy) and tended to have a similar distribution pattern as that detected in the HIo and AMLo groups with a more obvious difference in the CD8+ population (Figs. 1g and 2a, b). These findings indicate that the leukemia microenvironment might drive T cell differentiation in AMLy. Whether such a skewed T cell distribution in AMLy truly represents T cell senescence remains an open question [8]; however, T cells in AMLo patients may not be able to further differentiate due to inherent T cell senescence, which may be an immune factor underlying the inferior prognosis of AMLo patients. Together, these data may suggest that T cell exhaustion and senescence are involved in T cell immune impairment, leading to an inefficient anti-tumor response.

We next compared differences in the distribution of memory T cell subsets between the AMLy, AML-CR, and HIy groups. A persistent, skewed memory T cell distribution was demonstrated for AML patients who achieved CR after chemotherapy (Fig. 2c, d). CD4+ and CD8+ T_SCM_ cells were predominantly increased at different time points after CR, while the change in other memory T cell subsets was relatively different (Fig. 2e, f). Overall, with the exception of incomplete recovery of the T_SCM_ cells, the reduction in T_CM_ cells and corresponding excessive accumulation of T_EM_ and T_EF_ cells were more evident in AML patients with CR (Fig. 1g), which may be related to the immune suppression of chemotherapy.

## Abbreviations

AML: Acute myeloid leukemia
BM: Bone marrow
CML: Chronic myeloid leukemia
CR: Complete remission
HSCT: Hematopoietic stem cell transplantation
PB: Peripheral blood
PBMCs: Peripheral blood mononuclear cells
T_CM_: Central memory T cells
T_EF_: Terminal effector T cells
T_EM_: Effector memory T cells
T_SCM_: Stem cell memory T cells
